# Modeling Arboviral Infection in Mice Lacking the Interferon Alpha/Beta Receptor

**DOI:** 10.3390/v11010035

**Published:** 2019-01-08

**Authors:** Alejandro Marín-Lopez, Eva Calvo-Pinilla, Sandra Moreno, Sergio Utrilla-Trigo, Aitor Nogales, Alejandro Brun, Erol Fikrig, Javier Ortego

**Affiliations:** 1 Center for Animal Health Research, INIA-CISA, 28130 Valdeolmos, Madrid, Spain; alejandro.marinlopez@yale.edu (A.M.-L.); evacalvopinilla@gmail.com (E.C.-P.); moreno.sandra@inia.es (S.M.); sergioutrilla@gmail.com (S.U.-T.); aitor_nogales@hotmail.com (A.N.); brun@inia.es (A.B.); 2 Section of Infectious Diseases, Department of Internal Medicine, Yale University School of Medicine, New Haven, CT 06510, USA; erol.fikrig@yale.edu

**Keywords:** arbovirus, IFNAR(−/−) mice model, interferon, pathology, vaccines

## Abstract

Arboviruses are arthropod-borne viruses that exhibit worldwide distribution and are a constant threat, not only for public health but also for wildlife, domestic animals, and even plants. To study disease pathogenesis and to develop efficient and safe therapies, the use of an appropriate animal model is a critical concern. Adult mice with gene knockouts of the interferon α/β (IFN-α/β) receptor (IFNAR(−/−)) have been described as a model of arbovirus infections. Studies with the natural hosts of these viruses are limited by financial and ethical issues, and in some cases, the need to have facilities with a biosafety level 3 with sufficient space to accommodate large animals. Moreover, the number of animals in the experiments must provide results with statistical significance. Recent advances in animal models in the last decade among other gaps in knowledge have contributed to the better understanding of arbovirus infections. A tremendous advantage of the IFNAR(−/−) mouse model is the availability of a wide variety of reagents that can be used to study many aspects of the immune response to the virus. Although extrapolation of findings in mice to natural hosts must be done with care due to differences in the biology between mouse and humans, experimental infections of IFNAR(−/−) mice with several studied arboviruses closely mimics hallmarks of these viruses in their natural host. Therefore, IFNAR(−/−) mice are a good model to facilitate studies on arbovirus transmission, pathogenesis, virulence, and the protective efficacy of new vaccines. In this review article, the most important arboviruses that have been studied using the IFNAR(−/−) mouse model will be reviewed.

## 1. Introduction

Arboviruses are arthropod-borne viruses that exhibit worldwide distribution and are a constant threat, not only for the public health but also for wildlife, domestic animals, and even plants. The rise in global travel and trade as well as the changes in the global climate conditions are facilitating the expansion of the vector transmitters, including mosquitoes, ticks, sandflies, and midges among other arthropods, from endemic to new areas, augmenting the number of outbreaks around the world at an unprecedented rate. Arboviruses need multiple hosts to complete their cycle (i.e., host and vector), making it possible to impact disease by targeting either the arthropod vector and/or the pathogen. For some of these pathogens, efficient antivirals or vaccines are not available, in some cases due to the genetic variability of these viruses. Moreover, there are a limited availability of animal models to study infections, and some of them display a poor immunogenicity and some others viral infections cause neglected diseases that have not been deeply studied. Transmission between the vector and the host occurs when the vector feeds on the blood of the host by biting. However, the vector does not act as a simple vehicle that passively transfer viruses from one individual to another. Instead, arthropod-derived factors found in their saliva have an important role in infection and disease, modulating (positively and negatively) replication and dissemination within the host [1,2]. In addition, the inflammatory response that the host mounts against these vector molecules can enhance the severity of arbovirus infection [3,4].

To study disease pathogenesis and to develop efficient and safe therapies to prevent (vaccines) or treat (antivirals) viral infections, the use of an appropriate animal model is a critical concern. The use of mice as small animal models to study immunity, pathogenesis, as well as to test candidate vaccines and antivirals against a largely variety of viral diseases is widely spread. They are cost effective, being affordable for most of research laboratories. They reproduce quickly, are easy to handle, do not require specialized facilities to house, and multiple inbred strains of genetically identical mice are available. In many cases such as Crimean Congo Hemorrhagic Fever (CCHFV), Bluetongue (BTV), Middle East respiratory syndrome (MERS), or Ebola (EBoV) viruses, the pathogenesis of disease in humans is also partially mimicked. Furthermore, optimal reagents have been developed for in vivo and in vitro studies in mice, a fact which allows the study of other animal viruses apart from those which are human specific [5,6,7,8]. Also, it is possible to manipulate the mouse genome and generate transgenic, knock-out, knock-in, humanized, and conditionally mutant strains to interrogate protein function in physiological and pathological signs.

Immunocompetent wild-type mice are susceptible to infections with a number of viral pathogens such as influenza virus [9]; severe acute respiratory syndrome coronavirus (SARS-CoV) [10]; and Rift Valley fever virus (RVFV) [11]. Unfortunately, immunocompetent mice are not susceptible to many other viruses with outbreak potential, and thus alternative strategies are needed. 

## 2. IFNAR(−/−) Mice

In the early 90s, Muller and colleagues [12] generated mice deficient in the type I IFN (IFN-α/β) receptor (IFNAR(−/−)) by homologous recombination in embryonic stem cells. While these transgenic mice did not show any overt abnormalities by six months of age and were fertile, the animals were entirely unresponsive to the effects of type I IFNs. To monitor the response to type I IFN in vivo, they analyzed the induction of the *Mx-1* gene, a strictly type I IFN-specific response marker in mouse cells [13]. Mice infected with vesicular stomatitis virus (VSV), Semliki Forest virus (SFV), vaccinia virus (VV), or lymphocytic choriomeningitis virus (LCMV) showed a completely abrogated IFN type I response and an enhanced infection susceptibility, resulting in either higher viral organ titers compared to wild-type mice and death in case of VSV and SFV challenges [12]. 

**Figure 1:**
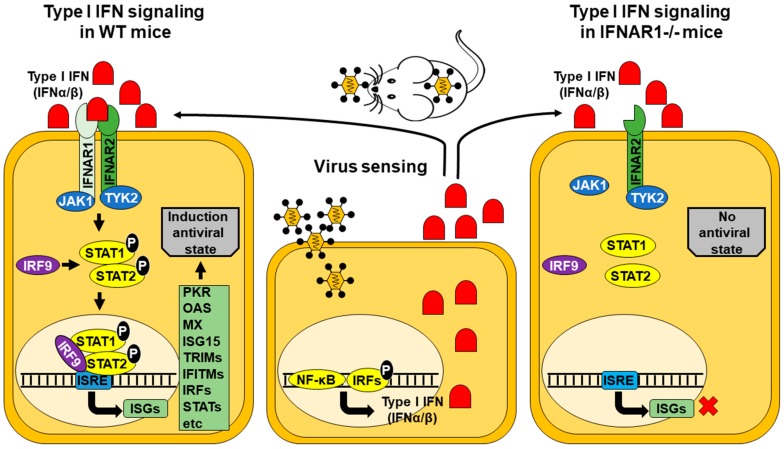
Signal transduction by the type I IFN receptors in wild-type (WT) or IFNAR−/− mice. Transcription of IFN genes is induced rapidly in response to viral infection. Cells sense viruses using multiple signaling pathways that ultimately will activate several transcription factors and their subsequent translocation into the nucleus, resulting in the activation of type I IFN (IFNα/β) genes. In WT mice, the released type I IFN is bound by the specific receptors IFNAR1/IFNAR2 trigging phosphorylation of JAK1/TYK2 kinases that activate STAT1 and STAT2. Phosphorylated STAT1/STAT2 heterodimers bind IRF9 and the complex is translocated to the nucleus where it induces expression of ISGs with ISRE-dependent promoters. The expression of ISGs will induce an antiviral state to prevent viral infection. However, in IFNAR1−/− mice, the antiviral state is not created, and cells are more susceptible to be infected. JAK, Janus activated kinase; TYK2, tyrosine kinase 2, ISRE, IFN-stimulated response element; ISG, IFN-stimulated gene; OAS, oligoadenylate synthetase; MX, myxovirus resistance; ISG15, IFN-stimulated gene factor 15; TRIM, tripartite motif-containing proteins; IFITM, IFN-induced transmembrane proteins; IRF, IFN-regulatory factors; STAT, signal transducer and activator of transcription; NF-κ B, nuclear factor of kappa light polypeptide gene enhancer in B-cells.

The role of interferons (IFNs) against viral diseases has been widely studied, as well as the strategies evolved by viruses to antagonize the effects of IFNs. Both type I and type II IFNs have been implicated in the host antiviral defense and in the immunomodulatory functions that are critical during virus infection, not only limiting virus replication and initiating an appropriate antiviral immune response, but to also negatively regulating this response to minimize tissue damage (Figure 1) [14,15].

Type I IFNs are well known for their ability to directly induce an antiviral response within infected and surrounding cells, displaying autocrine and paracrine activities through the upregulation of molecules that can antagonize with multiple stages of virus replication, as the interferon stimulated genes (ISGs). Nearly all types of cells are capable of producing IFN-α/β, which are the best-defined and most broadly expressed type I IFNs; however, during the course of an infection, specialized immune cells known as plasmacytoid dendritic cells (pDCs) produce the vast majority of IFN-α [16]. As they are produced rather early on during an infection, type I IFNs are also essential for activating the antiviral innate immune response, such as natural killer (NK) cell effector functions [17,18,19].

In addition to type I IFN effects related to the antiviral state and innate immunity activation, the IFN system is linked to a variety of effector responses of the adaptive immune systems. Cytotoxic T cells (CTLs) are one of the two major effector cell populations regulated by type I IFNs (with NK). Type I IFNs have been shown to facilitate cross-presentation by DCs of viral antigens to CD8+ T cells [20]. The recruitment of cytotoxic cells to the site of infection mediated by chemokine production has been shown, as well as the induction of cytokines from CTLs that positively regulate cytotoxic cell populations and activities, as interleukin (IL)-15 type I IFN-induced production, which plays a critical role in proliferation and maintenance of NK cells and memory CD8+ T cells [19]. On the another hand, some reports showed how IFN-I exert an antiproliferative effect on anti-CD3-stimulated CD4 T cells during in vitro culture [21,22], but the opposite result is found when IFN-I produced in response to LCMV immunization act directly on virus-specific CD4 T cells, contributing to their clonal expansion [23]. The role of type I IFN production during apoptosis has also been studied. Given that viruses require host cell machinery to replicate, elimination of the infected cell would shut down this machinery, preventing viral spread. It is known that cells treated with type I IFNs sensitizes them to apoptosis upon subsequent viral infection, and some mechanisms of sensitization have been elucidated, such those that involve PKR or p53 [24,25]. Type I IFNs are also important during neurotropic viral infections, where they play multifaceted roles at the blood brain barrier (BBB). Type I IFN treatment decreases BBB permeability, enhances tight junction (TJ) integrity, and restricts leukocyte migration across the BBB into the central nervous system (CNS) parenchyma [26,27,28]. The induction of type I IFN expression following detection of viral pathogens such as West Nile virus (WNV) acts directly on BBB endothelium to preserve the formation of TJ and limit BBB permeability, antagonizing with the effect promoted by Th1 cytokines also secreted during WNV infection [28]. 

The IFNAR(−/−) knock-out receptor mouse model has been used to study infection, disease, pathogenesis and vaccine testing against multiple arbovirus families such as Togaviridae, Bunyaviridae, Flaviviridae, Rhabdoviridae, Orthomyxoviridae, and Reoviridae (Table 1). In this review, animal arbovirus families known to have been studied using the IFNAR(−/−) mice model are mentioned, describing briefly some examples, in which different aspects of biology, immunology, pathology, and vaccine design against these pathogens are exposed.

## 3. Families Included in the Order Bunyavirales

This large and diverse group has been more formally organized (https://talk.ictvonline.org/files/ictv_official_taxonomy_updates_since_the_8th_report/m/plant-official/6694). It comprises ten different families that include segmented negative strand virus species infecting plants, arthropods, and vertebrates. This group includes tri-segmented negative-strand RNA viruses, commonly known as bunyaviruses of which several members are important pathogens of animals and humans.

### 3.1. Rift Valley Fever Virus (Family Phenuiviridae)

Rift Valley fever virus (RVFV), a phlebovirus transmitted mainly by *Aedes* (*Stegomya*) mosquitoes, causing Rift Valley fever, a zoonotic disease of ruminants, has been confined to Sub-Saharan Africa for many decades. In the last years, a spectacular increase in the number of outbreaks, including a more northward geographic spread has been documented. This zoonosis is associated with “abortion storms” in domesticated sheep flocks and high mortality rates in newborn livestock (lambs and calves) [70]. Rift Valley fever virus is one of the major public health threats in sub-Saharan Africa, where human infection leads to a wide spectrum of clinical signs and symptoms that range from a “flu-like” illness with fever and myalgia to severe encephalitis, retinitis, and fatal hepatitis with hemorrhagic fever (1–2% of the cases) [71]. The viral and host cellular factors that contribute to RVFV virulence and pathogenicity are still poorly understood. Although RVFV is able to infect and replicate in wild-type mice [72], some studies using the IFNAR(−/−) mouse model have been also performed to study the role of type I IFN signaling and the mechanism of RVFV to evade the IFN response during the course of the infection. Bouloy and colleagues brought to the light the ability of RVFV to inhibit IFN-α/β synthesis, demonstrating that IFN type I production correlates with virulence and suggesting that the accessory non-structural protein NSs is an IFN antagonist factor that prevents IFNs-α/β from being induced early during the course of RVFV infection. Also, these authors showed how two RVFV strains, MP12 and clone 13, are attenuated in immunocompetent mice and in IFN-γ receptor-deficient mice but fully lethal in IFNAR(−/−) mice [29]. These observations suggested the use of IFNAR(−/−) mice as a candidate model for testing the efficacy of experimental vaccines and/or therapeutics against RVFV under a BSL-2 containment environment, using these attenuated strains. Thus, the efficacy of DNA vaccines encoding different RVFV antigens was tested in this model, showing several degrees of protection upon a lethal challenge [30,31]. The antiviral activity of silver nanoparticles was also tested in these mice showing reduction of viremia and delayed mortality after lethal challenge [32]. Finally, an important aspect that arose from the efficacy studies of MVA-vectored RVFV vaccines in IFNAR(−/−) mice was related to the opposite efficacy outcomes observed in IFNAR(−/−) and wild-type mice, providing important clues to dissect the role of cell-mediated immune responses in protection [33].

### 3.2. Crimean Congo Fever Virus (Family Nairoviridae)

Another emerging pathogen with epidemic potential is Crimean Congo Fever Virus (CCHFV), typically spread by tick bites of the *Hyalomma* genus, or by contact with blood or tissues of infected livestock (whose are usually asymptomatic) or patients [73]. Susceptibility of wild-type 129 Sv/Ew and IFNAR(−/−) mice to CCHFV was studied, showing viremia and viral titer in several organs as spleen, liver, kidney, brain, and heart in both immunocompetent and immunocompromised mice, but with high viral burden and developing an acute disease with fatal outcome, with a profound liver affectation in the case of IFNAR(−/−) mice [34]. In this mice, disease progression closely mimics hallmarks of human CCHF disease as marked proinflammatory host responses, severe thrombocytopenia and coagulopathy, making IFNAR(−/−) mice a good model to assess medical countermeasures [5]. Among them, formali-inactivated cell culture CCHF alum-adjuvanted vaccines, VLPs, DNA or viral vector vaccines (modified vaccinia virus Ankara (MVA) and adenovirus) expressing nucleocapsid protein or glycoproteins conferred different rates of protection in immunized animals [35,36,37,38,39] and administration of *Favipiravir* after infection (twice daily) suppressed the infection and the clinical signs in treated mice [40].

### 3.3. Schmallenberg Virus (Family Peribunyaviridae)

One non-zoonotic virus of this group with outbreak potential among domestic animals is Schmallenberg virus (SBV). The SBV causes congenital malformations and stillbirths in cattle, sheep, goat, and possibly in alpaca. Schmallenberg virus infection of susceptible pregnant animals can be associated with musculoskeletal and central nervous system malformations in stillborn or newborn lambs and calves [74]. Schmallenberg virus has spread throughout the European continent, spanning from Ireland to Turkey [75], since its discovery in Germany in 2011, and it has been shown to be transmitted by biting midges. Its close relation to Akabane virus (AKAV) suggests that much of what is known about that virus might also be applicable to SBV. Akabane virus propagation in mice was first described in 1976 [76], but it requires the intracranial injection of newborns. It has been shown that 2, 10, and 18-day old newborn NIH-Swiss mice intracerebrally inoculated with 400 plaque-forming units (PFUs) of SBV are also highly susceptible to the infection, with a 100% mortality rate, but this inoculation route does not resemble the natural route of infection [77]. In 2012, IFNAR(−/−) mice were shown to be susceptible to SBV infection, although clinical signs were not as evident. After SBV infection, mice showed primarily decreased weight loss, ataxia, apathy, but limited mortality [41]. In recent studies, it has been demonstrated that SBV virulence occurs as early as three days post-infection (dpi) and it becomes more severe at day six post-infection as observed by the significant weight loss and viremia [42]. The relation between type I IFN and viral spreading has been investigated using seve day old IFNAR(−/−) mice intracerebrally injected with SBV and an NSs deletion mutant, confirming the role of the NSs protein as a modulator, at least indirectly, of the IFN response in vivo [77]. This mouse model has been used to validate attenuated strains as potential vaccines [41], and to test protective immunity induced by the Gc-ecto1 domain and nucleocapsid protein of the virus, showing that these could be valid candidates for the development of subunit vaccines [42]. Wernike et al. [43] have also demonstrated the suitability of using Gc as an efficient vaccine in this murine model.

Other viruses of this family with remarkable impact in human health and livestock have been studied using this murine model. For instance, severe fever with thrombocytopenia syndrome virus (SFTSV) (with case fatality rates up to 30% in humans), Bunyamwera virus (BUNV), Dugbe virus (DUBV), and the Simbu virus (SIMV) [43,78,79,80,81], where the effect of the host IFN system and IFN-related genes on the outcome of infection, model suitability, and vaccination have been investigated in IFNAR(−/−) mice. 

## 4. Family Flaviviridae

Flaviviridae are a family of positive, single-stranded, enveloped RNA viruses. They are transmitted by mosquitoes and ticks and cause morbidity and mortality throughout the world. Some of them are known to produce hemorrhagic diseases, such as Dengue Fever virus (DENV), Yellow Fever virus (YFV), and Zika virus (ZIKV), and other members are also responsible of encephalitis diseases: West Nile virus (WNV), Japanese encephalitis virus (JEV), Powassan virus (PV), Langat encephalitis virus (LGTV) or Tick-borne Encephalitis (TBE).

### 4.1. Dengue Virus

Maybe the best-known and most widespread member of this family is dengue virus. Dengue Fever virus is the etiologic agent of the self-limited febrile illness dengue fever (DF), as well as the potentially lethal severe dengue disease (dengue hemorrhagic fever and dengue shock syndrome, DHF/DSS). Symptomatic infections are characterized by: fever, retro-orbital headache, muscle, joint and bone pain, nausea, vomiting, abdominal pain, mucosal bleeding, and thrombocytopenia. In the most severe form of the disease, severe bleeding, organ dysfunction, vascular permeability, and shock can occur. Replication of DENV has been tested in immunocompetent mice [82]. C57BL/6 mice infected with DENV-1 strain Mochizuki presented some signs of dengue disease such as thrombocytopenia, hemorrhage, liver damage, and increase production of IFNγ and tumor necrosis factor alpha (TNFα) cytokines. However, no changes in CD4 and CD8 populations were observed comparing infected and mock infected groups. In addition, this strain was propagated in newborn (1 to 2 days old) Swiss mice, by intracerebral (ic) inoculation of infected cell culture supernatant. This propagation method resulted in a neurological disease phenotype that is unlike the multi-organ involvement typically observed in clinical dengue infections [44]. Although this DENV strain induce detectable viremia in C57BL/6 strain, the overwhelming majority of immunocompetent mouse models do not result in clinical signs of dengue infection [44]. To overcome this issue, a pathological analysis were performed in IFNAR(−/−) mice. It has been shown that mortality rates depend on the DENV serotype and strain used [44]. A severe dengue-like disease is observed when animals are infected with sufficiently high DENV2 challenge doses and clearance of DENV from the central nervous system (CNS) and prevention of paralysis in this mouse model has been confirmed to be dependent of CD8+ T cells and IFN-γ response [45]. Most primary DENV infections with any serotype are asymptomatic or lead to the self-limited febrile illness DF, in patients infected with DENV. However, secondary infection with a different DENV serotype leads to increased risk of developing severe dengue disease [47]. This increase in severity upon secondary infection is thought to be mediated in part via antibody-dependent enhancement (ADE), whereby interaction between antibodies generated during a prior infection and the current infecting serotype can lead to increased uptake of virus via Fc receptors expressed on susceptible myeloid cells [46]. This phenomena was observed also in IFNAR(−/−) mice, with a dramatic increase in the mortality rate in individuals intraperitonially (ip) injected with anti-E mAb 4G2 24 h before challenge [48]. Additionally, another study published in 2009 revealed the important role for CD8+ T cells in the host defense against DENV, demonstrating that the anti-DENV CD8+ T cell response can be enhanced by immunization. This study identified DENV-specific CD8 T cell epitopes, and peptide vaccination with these epitopes resulted in enhanced control of DENV infection and viral load [83]. Another immunization study has been performed in this model using live attenuated dengue vaccine 2′-o-methyltransferase mutants, eliciting a strong adaptive immune response [84].

### 4.2. Yellow Fever Virus

Yellow Fever virus produced one of the most dangerous infectious diseases of the 18th and 19th centuries, resulting in mass casualties in Africa and the Americas [85]. Inoculation of wild-type 129 mice subcutaneously (sc) in each rear footpad with 10^4^ PFU of YFV did not result in any weight loss or death, whereas challenged 3–4 week old IFNAR(−/−) mice (129 background) challenged with YFV strains Asibi or Angola73 developed disease under the same conditions. During infection, non-structural protein 5 (NS5) protein inhibits IFN signaling by binding to STAT2 protein and promoting its degradation [86]. In mouse infection, NS5 was not able to bind murine STAT2, allowing IFN-mediated clearance of the virus. The IFNAR(−/−) mice were shown to be susceptible to the challenge, with death occurring between 7–9 dpi. Additionally, the mice developed viscerotropic disease with virus dissemination to the visceral organs, spleen, and liver, in which severe damage can be observed with gross pathological examination and hematoxylin/eosin staining. Moreover, elevated levels of MCP-1 and IL-6 in these organs were detected, suggesting an unleashing of “cytokine storm” [49].

### 4.3. Zika Virus

Another member of this family with high outbreak potential is Zika virus. Zika virus infections in humans have sporadically occurred in Africa and Asia, and new outbreaks were registered in small island countries located in the Pacific Ocean, such as Yap Island [87], French Polynesia [88], and Easter Island [89]. In 2015, an epidemic of ZIKV originating from Brazil, spread through most of North and South America and the Caribbean, as well as thousands of imported cases from travelers returning to their home countries after visiting outbreak areas [90,91,92]. The ZIKV infections are typically asymptomatic, but in some cases the disease courses with fever, joint pain, maculopapular rash, and red eyes [93]. While no deaths have been reported from ZIKV infections, mother-to-child transmission during pregnancy may result in congenital Zika syndrome with abnormalities in the central nervous system (microcephaly, intellectual development, seizures, and vision impairment) [94]. Zika virus infections in adults is associated with Guillain–Barré syndrome [95]. Distinct from other flavivirus infections, sexual transmission of ZIKV from male-to-male, male-to-female, and female-to-male have been documented [96,97,98,99]. Wild-type mice are refractory to Zika infection with strain MP1751 (Zika virus targets human STAT2 to inhibit type I interferon signaling, but not murine STAT2 [55], as was observed with YFV), while IFNAR(−/−) mice succumbed to disease at 6 dpi with 20% body weight loss with a challenge of 10^6^ PFU sc. Viral RNA was observed at 3 and 7 dpi in blood by RT-qPCR, as well as high levels of virus in spleen, brain, ovary, and liver of these animals. Pathology studies show that inflammatory and degenerative changes could be detected in the brain [50]. More studies have been performed using alternative strains/doses and different ages, as H/PF/2013 strain from French Polynesia and the original Ugandan ZIKV strain MR 766. Five- to 6-week-old mice sc infected with 10^2^ focus-forming units (FFUs) began to lose weight by five days after infection, and by day seven, when they began to succumb to infection, animals had lost between 15% and 25% of their starting body weight. Ten and 13 days after infection, mice exhibited 100% and 80% lethality with ZIKV H/PF/2013 and MR 766, respectively. When mice were challenged intravenously, an increase of 60% in the survival rate was observed in MR 766 infected mice [51]. In older IFNAR(−/−) mice (3-, 4-, and 6-month-old), infection with 10^3^ FFU of ZIKV (H/PF/2013) reduced the weight in all animals, with ∼30% of starting weight lost by nine days after infection, and a mortality of 60–20% were observed [51]. Interestingly, the lethality in 10–12-week-old animals was abolished when using 10^5^ PFUs of ZIKV FSS13025 strain from Asian lineage (being a 100% and 50% of lethality in 3- and 5-week-old mice) [52], but not for ZIKV H/PF/2013 infection (100% of deaths) [53]. Taken together, these results indicate that the disease caused by ZIKV infection in these animals was age and strain-dependent. Surprisingly, another strain associated with microcephaly case, ZIKV-Paraiba, caused weight lost in 5–8-week-old IFNAR(−/−) mice inoculated sc with 10^2^ or 10^4^ PFUs at days 6 and 7 post-infection, independent of the dose of ZIKV [53]. Approximately 50% of the mice succumbed to disease or were euthanized between days 9 and 11 due to development of neurological signs such as hind limb paralysis. Viral RNA was detected in many tissues as mandibular lymphonode, salivary gland, lung, heart, liver spleen, kidney, bladder, gonad, spinal cord, brain, cerebellum, and blood at different time points (3 and 8 days). The route of inoculation does not seem to be significant among subcutaneous, intraperitoneal, and footpad administration, but in this study, only ip resulted in uniform lethality in young IFNAR(−/−) mice [53]. Sexual and maternal transmission are the most important concerns in Zika disease due to the consequences derived of ZIKV infection in the fetus. In IFNAR(−/−) males, high levels of viral RNA and antigen within the epididymal lumen (where sperm is stored) and within surrounding epithelial cells was observed. Moreover, serum testosterone levels were markedly decreased at 8 dpi and also observed was a reduction in the size of the testes at 21 days post-infection [100,101]. In females, vaginal infection with high doses of ZIKV was lethal. Vaginal ZIKV infection of pregnant female mice at various gestational time points led to fetal growth restriction. High levels of local ZIKV replication were observed starting on 2 dpi, and ZIKV continued to replicate in the vaginal tissue through 7 dpi, suggesting that type I IFN play a critical role in blocking ZIKV replication in the vaginal mucosa [102]. The role of type IFN I during pregnancy in infected mothers have been assessed using IFNAR(−/−) mice [103], and the findings highlight the detrimental impact of type I IFN on the developing placenta and fetus by demonstrating that only the fetuses with a functional copy of IFNAR are resorbed after ZIKV infection, whereas their IFNAR(−/−) littermates continue to develop, even having higher ZIKV titers in their placentas. These results implicate type I IFNs as a possible mediator of pregnancy complications, including spontaneous abortions and growth restriction, in the context of congenital viral infections. New generation vaccines have been shown effective against ZIKV in IFNAR(−/−) mice based on VSV viral vector expressing pRM and E ZIKV proteins, enhancing ZIKV-specific IgG with neutralizing activity, and providing protection within three days of vaccination [104]. A vaccinia-based single vector that encodes the structural polyprotein cassettes of both Zika (and chikungunya) viruses from different loci has also been recently developed. A single vaccination of mice induces neutralizing antibodies and prevent viremia and fetal/placental infection in female IFNAR(−/−) mice and testes infection and pathology in male IFNAR(−/−) mice [105]. The IFNAR(−/−) mice model has also been used to demonstrate how salivary factors expressed by the vector *Aedes aegypti* modulates ZIKV infectivity. Administration of the salivary factor LTRIN caused a substantial loss in body weight in IFNAR(−/−) mice up to 10–15% of their starting body weight by day 6, indicating that the administration of LTRIN exacerbated ZIKV’s pathogenesis in IFNAR(−/−) mice [106]. 

### 4.4. West Nile Virus and Japanese Encephalitis Virus

West Nile virus (WNV) is generally transmitted by *Culex* mosquitos and the natural host are birds. In addition, bites from infected mosquitos can infect humans and other mammals as horses. However, they are “dead end” hosts because they do not develop high levels of virus in their bloodstream, and cannot generally pass the virus on to other biting mosquitoes [107]. West Nile virus is endemic in Africa, Asia, Europe, and Australia, and has spread into Canada and the United States (U.S.) [108]. West Nile virus infection of humans can be characterized as asymptomatic or as a mild, febrile illness termed West Nile fever. However, a significant increase in the global incidence of severe neurological disease (associated with WNV lineage I infections) arose in the mid-1990s, culminating in the U.S. outbreak in 2003, which included 9862 reported cases and 264 deaths [109]. After its introduction in New York City in 1999, WNV rapidly spread across the continent and now appears to have firmly established itself in the ecology of North America. The rapid emergence of WNV and its virulence within a naïve population suggest that epidemic forms of the virus may encode mechanisms to evade host immunity [110].

West Nile virus is known to cause disease and death in wild type mice, but studies using IFNAR(−/−) mice have been performed to elucidate the early mechanisms in the IFN immune response. In this study, the authors showed the high susceptibility of IFNAR(−/−) mice to WNV infection. The 8–10 week old IFNAR(−/−) mice (129Sv/Ev background) challenged with 10^0^, 10^1^ or 10^2^ PFU (strain 3000.0259) via footpad inoculation showed severe clinical symptoms by 3 dpi, including hunched posture, ruffled fur, and reduced activities, regardless of dose. Death (100%) occurred within 12–48 h after the onset of symptoms, and the mean time to death was 4.6 ± 0.7 and 3.8 ± 0.5 dpi for IFNAR(−/−) mice in the 10^0^ and 10^2^ PFU groups, respectively. Infectious virus was detected in the muscle, heart, lung, kidney, and liver [56]. Also, an altered cellular tropism was observed in IFNAR(−/−) mice, with increased infection in macrophages, B cells, and T cells in the spleen, compared with wild-type mice [56]. Another feature of WNV is its capability of infecting the CNS, causing fatal encephalitis. In vivo, IFNAR(−/−) mice exhibited enhanced BBB permeability and TJ dysregulation after WNV infection, triggered by pattern recognition receptors-mediated cytokine expression. These results suggest that local CNS type I IFN responses may act on the BBB to mitigate the access of WNV to the CNS parenchyma [28]. Regarding vaccine development in this model, a novel single-cycle flavivirus vaccine has been tested, with a significant increase in the level of WNV-specific CD8+ T cells compared to the wild-type [111].

Japanese encephalitis, whose causal agent is JEV, is considered as one of the most important encephalitic arthropod-borne diseases. An estimated 3 billion people live in countries where the disease is endemic and 30,000–50,000 cases and 10,000–15,000 deaths are reported annually [112,113]. Wild-type mice are susceptible to the sc JEV infection, with survival rates that vary between 10–40%, being not dose-dependent [57]. In the same study, inoculations were repeated in 5–6 week old IFNAR(−/−) mice at the same doses, being highly susceptible to the challenge, with uniform, dose-dependent death occurring between 64–120 h. Viral replication could be detected in the spleens and brains of infected animals, with peak titers at 48 h [57].

Other flaviviral encephalities are being studied using this mouse model of infection, as that caused by Langat encephalitis virus (LGTV), showing type I IFN as a critical factor to control LGTV infection, as LGTV RNA was found in all organs in the absence of IFNAR, whereas in wild-type mice only low viral burdens can be detected in the olfactory bulb [114,115].

## 5. Family Togaviridae

The Togaviridae family are composed for linear, non-segmented, single-stranded, positive sense RNA viruses. Among this family, only the genus *alphavirus*, are transmitted by arthropod vectors. Sindbis, Semliki, chikungunya, Mayaro, O’nyong-nyong or Ross River alphaviruses are known to cause human diseases in which rheumatic complaints are a major feature, while eastern equine encephalitis, and Venezuelan equine encephalitis viruses can cause arthritis disease and encephalomyelitis, a potentially fatal inflammatory disease of the CNS with frequent long-term neurological deficits in survivors [116,117,118,119,120].

### 5.1. Chikungunya Virus

Wild-type C57BL/6 mice infected with 10^4^ cell culture infectious dose 50 (CCID_50_) of CHIKV (Asian or the Reunion isolates) produced a measurable self-limiting perimetatarsal foot swelling with clear histological signs of acute and persistent inflammatory disease [121]. In IFNAR(−/−) mice, the susceptibility as well as the role of IFNAR receptors in CHIKV control and clearance have been studied. A dose of 10^2^ PFU injected intradermally (id) was sufficient to kill the IFNAR(−/−) mice between days 2.5 and 4 post-infection, and injection of 10^6^ PFU resulted in even faster death, with all animals succumbing to infection between days 2–3 post-infection [122]. Similar to what was observed in highly viremic humans [123], the viral load in IFNAR(−/−) mice infected with 10^6^ PFU at 2 dpi was >10^8^ tissue culture infectious dose 50 (TCID_50_)/mL. In contrast, wild-type animals cleared the infection with undetectable serum viral titers at all timepoints tested. CHIKV exhibits a marked tropism for skeletal muscles, joints and skin, which constitute the classical symptomatic organs in the human disease. Fibroblasts constitute the principal CHIKV cell target in all these organs. Before reaching its target organs, CHIKV undergoes an early burst of viral replication in the liver, where CHIKV antigens are primarily detected in sinusoidal capillary endothelial cells and to a lesser extent in Kupffer cells. At 3 dpi, there is a sharp increase in viremia, with CHIKV antigens detectable in the red pulp of the spleen. In the case of severe CHIKV infection, CHIKV disseminates to the CNS, as is observed in human [124], via the choroid plexus route, and undergoes viral replication at the ependyma and leptomeningeal levels, not being detected at the brain micro-vessel and parenchyma. Maternal–fetal transmission of CHIKV in pregnant IFNAR(−/−) mice has also been analyzed. However, CHIKV is unable to cross the placental barrier from the mother to the fetus in the mice [58] and humans, with some exceptions, as the three cases reported in the second trimester of gestation, which CHIKV infection has been associated with antepartum fetal deaths without clear evidence for the mechanism [125].

Some vaccines and therapeutic measures against CHIKV infection have been evaluated in this model. Mouse anti-CHIKV monoclonal antibodies (MAbs), selected for their ability to inhibit infection of all three CHIKV genotypes, have been tested using IFNAR(−/−) mice. Four neutralizing MAbs (CHK-102, CHK-152, CHK-166, and CHK-263) that have been mapped to distinct epitopes on the E1 and E2 structural proteins, provided complete protection against a lethal challenge. CHK-15, the most protective MAb, was humanized, shown to block viral fusion, and require Fc effector function for optimal activity in vivo. In post-exposure therapeutic trials, administration of a single dose of a combination of two neutralizing MAbs (CHK-102 + CHK-152 or CHK-166 + CHK-152) limited the development of resistance and protected immunocompromised mice against disease when given 24 to 36 h before CHIKV-induced death, so the use of these highly neutralizing MAbs may be a promising treatment option for CHIKV in humans [126].

A vaccine based on a chimeric VSV that expresses the CHIKV envelope polyprotein (E3-E2-6K-E1) in place of the VSV glycoprotein (G) and also expresses the membrane-envelope (ME) glycoproteins of ZIKV has been generated. This vaccine induced neutralizing antibody responses to both CHIKV and ZIKV in IFNAR(−/−) mice, conferring protection against both pathogens just with a single vaccination [127]. An insect-specific alphavirus, Eilat virus (EILV), has been used as a vaccine platform to generate EILV/CHIKV chimeras expressing CHIKV structural proteins, and is structurally identical to wild-type CHIKV virus. The replication-defective nature of EILV/CHIKV in vertebrate cells, despite its ability to replicate to exceptionally high titers in insect cells, elicited rapid (within four days) and long-lasting (>290 days) neutralizing antibodies that provided complete protection in IFNAR(−/−) mice. This platform represents the first structurally native application of an insect-specific virus in preclinical vaccine development and highlights the potential application in the development of vaccines against other arboviruses [128].

### 5.2. Other Alphavirus

The role of the IFNAR receptor has been assessed in other members of this family. The prototypic alphavirus, Sindbis virus strain AR339, was isolated by ic inoculation of three-day-old mice with a mosquito homogenate collected near Sindbis, Egypt. In wild-type mice, the infection courses asymptomatic, while IFNAR(−/−) mice inoculated sc with 10^2^ PFUs of TR339 succumbed to the infection within 3–4 dpi. By 24 hpi, a high-titer serum viremia had seeded infectious virus systemically, coincident with the systemic induction of the proinflammatory cytokines IL-12 p40, IFN-gamma, TNFα, and IL-6. Replicating virus was located in macrophage-dendritic cell (DC)-like cells at 24 hpi in the draining lymph node and in the splenic marginal zone. By 72 hpi virus replication was widespread in macrophage-DC-like cells in the spleen, liver, lung, thymus, and kidney and in fibroblast-connective tissue and periosteum, with sporadic neuroinvasion. Thus, type I IFN protects the normal adult host from viral infection by rapidly conferring an antiviral state on otherwise permissive cell types, both locally and systemically. Ablation of the type I IFN system alters the apparent cell and tissue tropism of the virus and renders macrophage-DC-lineage cells permissive to infection [59]. IFNAR(−/−) mice infected with Venezuelan equine encephalitis (VEE) also exhibit progressively increasing signs of infection characterized by pronounced hunching, lethargy, prostration, and death. Accelerated VEE dissemination to serum, spleen, and brain was observed in these mice compared with wild-type animals, and is associated with the upregulation of proinflammatory genes [60]. O’nyong-nyong (ONNV) infected mice exhibited 50–55% mortality after a sc dose of 10^3^ PFU. Mortality increased to 100% when the ONNV dose was increased to 10^4^ PFU. The ONNV was present in the brain and skeletal muscle of IFNAR(−/−) mice, and the presence of virus in the heart could be a function of myocyte tropism as has been reported in CHIKV infection [129]. It is of interest that the inflammatory infiltrate seen in the tissues of mice was composed predominantly of monocytes and myositis/tenosynovitis, but not the neurologic disease was observed in infected animals. In addition, IFNAR(−/−) mice generated a viremia peaking on days 2–3 post-infection that waned by day 5, which is typical of alphavirus infections in humans.

## 6. Family Rhabdoviridae

Rhabdoviridae is a virus family with a very broad host range that are capable of infecting plants, and invertebrate and vertebrate animals. Rhabdoviruses have a non-segmented, linear, negative-sense, single-stranded RNA genome. This RNA molecule codes for five viral proteins and its complete genome is approximately 11 kbp–15 kbp. Rhabdoviridae contains six genera: *Lyssavirus*, *Ephemerovirus*, *Norvirhabdovirus*, *Cytorhabdovirus*, *Nucleorabdovirus*, and *Vesiculovirus*, being the last the only transmitted by arthropods in animals. The prototype of *Vesiculovirus* genus is VSV, an arthropod-borne virus that primarily affects rodents, cattle, swine and horses. It can induce mild symptoms upon infection in humans and other species and may also cause severe foot- and mouth-like disease in cattle and pigs. Vesicular stomatitis virus replicates rapidly, developing high levels of progenies in a minimum amount of time and strongly interferes with the host’s cell metabolism. Infection by rhabdoviruses induces a cellular response through the activation of pattern recognition receptors (PRRs) that causes the production and secretion of IFN and pro-inflammatory cytokines. The virus replication is highly sensitive to the inhibitory action of IFN therefore IFNAR(−/−) mice are highly susceptible to VSV pathogenesis [61,62]. Interferon plays a critical role for virus control after a VSV infection, although the concrete mechanisms are unknown. Several studies have been carried out in IFNAR(−/−) mice to discover these mechanisms and whether IFN expression play a role in determining viral tropism. In fact, a study carried out by Detje et al. [61] has shown that IFN triggering within the periglomerular cells of the olfactory bulb is required to protect against lethal disease.

## 7. Family Orthomyxoviridae

Orthomyxoviridae is a family of enveloped viruses, generally rounded but that can be filamentous. Eight ssRNA segmented and negative-sense linear molecules compose its genome (13.5 Kb), which is encapsidated by a nucleoprotein (NP) constituted layer and encodes 11 proteins. They present a global distribution, are more common in winter, and they are characterized by causing an acute infection of the respiratory tract. Within this family are the genera *Influenza virus (type A, B, C, and D)*, *Thogotovirus*, *Isavirus*, and *Quaranjavirus*, where *Thogotovirus* and some species of *Quaranjavirus* are the unique genus transmitted by arthropods (mainly ticks) within this family.

Thogoto virus (THOV), is the prototype of tick-transmitted orthomyxoviruses and shares structural and genetic similarities with its relative, influenza virus. In contrast to influenza virus infection, which is mediated via the respiratory system and thus acting locally, THOV, as a tick-mediated virus, is acting systemically. Moreover, for THOV but not influenza virus, mice are an important natural host [130,131]. It has been shown that THOV induces type I IFN responses in several cell lines and mouse embryonic fibroblasts [132,133] in vivo. Using the IFNAR(−/−) mice model, it has been possible to determine how THOV infection of mice leads to an unexpected strong and long-lasting mode of type I IFN expression that is most likely dominated by IPS-1-dependent IFN production of infected myeloid dendritic cells (mDC), but not plasmacytoid pDC cells [64]. Using replication-incompetent THOV-derived virus-like particles, the authors demonstrated that an infected host can use alternative pathways to induce type I IFN responses, independently of type I IFN receptor, induced by viral polymerase activity, but being largely independent of viral replication. This fact has an important relevance to understand how type I IFN can be produced in large amounts in specialized cell types independently of the IFNAR-dependent enhancement, broaden our view of host strategies to fight viral pathogens [63].

## 8. Family Reoviridae

The members of the genus *Orbivirus*, within the family Reovidae, can infect a wide range of hosts such as equids, ruminants, camelids, marsupials, seabirds, batsm and in some cases humans. The more relevant orbiviruses in animal health are Bluetongue virus (BTV), African horse sickness virus (AHSV), and Epizootic hemorrhagic disease virus (EHDV).

### 8.1. Bluetongue Virus

Bluetongue virus is the type species of this genus that can cause a severe hemorrhagic disease in ruminants, particularly in sheep. Other susceptible species are camelids and alpacas. Bluetongue virus has been responsible for important outbreaks all over the world affecting sheep, cattle, and deer, and resulting in huge economic losses. The study of many aspects of BTV infection and the evaluation of vaccines has long been hampered by the lack of a small animal model that supports this virus. While BTV is lethal in newborn mice, two-week old mice are largely refractory to infection. The first characterization of BTV infection in IFNAR(−/−) mice was developed after inoculation with serotypes 4 and 8 [6]. Afterwards, multiple serotypes and strains have been demonstrated to induce clinical signs, viremia, and mortality in this mouse model. The IFNAR(−/−) mice with a C57BL/6 and 129Sv/Ev genetic background exhibit the same level of susceptibility to BTV infection and no differences are found between subcutaneous and intravenous administration in the survival rates and appearance of disease [134,135]. The clinical manifestations that are found in IFNAR(−/−) mice inoculated with a lethal dose of BTV comprise ocular discharges, apathy, an increased respiratory rate and hunching [6]. Notably, these are some of the clinical signs, among others, that BTV infected ruminants may display [136]. Studies of viral progression in IFNAR(−/−) mice showed that infectious virus is recovered from the spleen, lung, thymus, lymph nodes, and blood. Thus, BTV disseminates via blood and lymph as it does in the natural hosts [6]. In mice, infected thymus exhibits a profound lymphoid depletion, a loss of thymic architecture as the medulla and the cortex are hardly distinguishable and large areas of the parenchyma with necrosis. In addition, a severe distortion of normal histology together with lymphoid depletion are observed in lymph nodes [6,66]. When virus infects spleen in IFNAR(−/−) mice, this shows a marked lymphoid depletion with severe white pulp lymphocytolisis and infiltration of neutrophilic infiltrates in the margin between the red and white pulp [66]. In these studies, a reduction in CD3 and CD79 (T and B cell markers, respectively) reactivity was observed in the spleen and thymus of BTV-infected mice that confirms the lymphopenia. This has been described commonly in BTV-infected sheep [137,138]. Moreover, lungs from infected mice reveal a diffuse interstitial pneumonia with hyperemia, increased septum size, a moderate edema in the alveolar cavity and infiltration of lymphocytes, macrophages, and neutrophils [66]. All these data indicate that the lesions found in BTV-infected IFNAR(−/−) mice are similar to those found in the natural hosts [65,139]. Changes in hematology including thrombocytopenia, neutrophilia, and lymphopenia have been determined after infection of IFNAR(−/−) mice with a high virulent strain of BTV-4 [66], observations similar to those described in experimental BTV infections [65,140].

Furthermore, this mouse model has been used to study the determinants of virulence of BTV field strains. Viruses were maintained in cell culture at low or high passage number and its virulence were evaluated in IFNAR(−/−) mice. The low passaged viruses BTV-2 and BTV-4 were lethal for mice, while the viruses that were extensively passaged become attenuated [141]. Interestingly, BTV-9 with a small number of passages were less pathogenic than the other strains tested, which correlates with the lower morbidity and mortality of this strain circulating in Italy in the early 2000s. Other studies compared the different degree of virulence in IFNAR(−/−) mice between a North European BTV-8 strain (BTV-8NET2006), that were highly virulent in the field, and a BTV-8 strain isolated in Italy in 2008 (BTV-8IT2008) that did not caused clinical signs. Experiments in mice reveal that mice inoculated with BTV-8NET2006 succumbed earlier to the infection than BTV-8IT2008 infected mice [142]. These data in a whole indicate that IFNAR(−/−) mice could be an adequate animal model to investigate the determinants of BTV virulence, factors of host interaction and pathogenesis.

A number of experimental vaccines for BTV have been tested in the mouse model based on IFNAR(−/−) mice. First characterization of these kind of vaccination trials was done using a commercial inactivated vaccine that has been used in the field, demonstrating that this vaccine prevent clinical disease in IFNAR(−/−) mice as it does in the natural host [6]. Then, the efficacy of novel recombinant subunit, DNA, and viral vector vaccines have been tested in the IFNAR(−/−) mouse model (Table 2).

### 8.2. African Horse Sickness Virus and Epizootic Hemorrhagic Disease Virus

African Horse Sickness Virus caused a severe disease in equids, where mortality could reach 90% in susceptible horses. Dogs can be also infected after feeding contaminated horsemeat and experimental infections have been established. On the other hand, a neurotropic vaccine strain can cause encephalitis and retinitis in humans, although no infections after contact with field strains have been described [155].

Earlier attempts to develop a mouse model to evaluate vaccines for AHSV were not successful using BALB/c mice, since AHSV vaccine strains were in most cases more virulent for mice that the wild-type strains [156]. Another study determined that although sc inoculation did not cause disease, intranasal inoculation of AHSV in immunocompetent mice increase the clinical fatality [157]. This could be explained by two hypothesis, the neurotropism acquired after intracerebral passages in mice [156] and the retrograde neuroinvasion through the olfactory pathway [158]. Nevertheless, a sc infection is a more similar route of inoculation comparing with the bite of Culicoides midges in nature and IFNAR(−/−) mice inoculated sc with AHSV are highly susceptible to the virus. Thus, this mouse model has been used to study virulence, pathology, and to evaluate vaccines with satisfactory results. 

Initial studies in IFNAR(−/−) mice were done by Castillo-Olivares et al. in 2011, describing that the pathology of the AHSV infected mice closely resemble those found in AHSV-infected horses with the exception of brain lesions [67]. This certain level of neutrotropism has also been observed in other studies [157] and may be resulted from the isolation of AHSV viruses in the brain of suckling mice. In IFNAR(−/−) mice infected with AHSV-4 (Madrid/1987), hemorrhages and inflammatory changes in the lung, splenomegalia, and congestion of other internal organs such as the liver were observed [67], and these are common pathological findings in horses infected with AHSV [159]. In addition, high expression of AHSV antigens was found particularly in spleen by immunofluorescence techniques. Further experiments by our group revealed the presence of high viral loads in spleen, thymus, liver, and lungs (data not published).

Differences in virulence between AHSV strains are present in nature and have also been observed in IFNAR(−/−) mice. Studies in IFNAR(−/−) mice using AHSV serotype 9 (PAKrrah/09) did not cause mortality with a dose of 10^6^ PFUs as occurred with same dose of AHSV-4 (Madrid/1987). Nevertheless another strain of AHSV-9 caused 33% of mortality with that infectious dose [157]. Although the infection of mice with ASHV-9 (PAKrrah/09) is not fatal in IFNAR(−/−) mice, clinical signs and viremia are present in the animals. The level of viremia was similar in animals infected with serotypes 4 and 9; however, the period of viremia was shorter when animals were infected with serotype 9 [68]. Recent studies to characterized AHSV serotype 3, revealed a higher virulence in the mouse model, since a low dose of 10^2^ PFUs per mouse killed all animals (unpublished data). Studies comparing other serotypes showed that mice infected with AHSV-4 had significantly higher AHSV RNA levels than mice infected with AHSV-1, suggesting that AHSV1 represents a less virulent serotype [160].

Several vaccine approaches have been evaluated against AHSV in IFNAR(−/−) mice and its efficacy compared to horses in some cases. The MVA vector expressing AHSV proteins has been widely studied. Immunization with MVA-VP2 stimulated neutralizing antibodies and showed protective capacity against homologous AHSV first in mice [67] and also in horses [161]. As well, the IFNAR(−/−) mouse model has been used to characterize the acquired immune responses of MVA-VP2 through the transfer of sera or splenocytes to recipient mice [162,163]. In other studies, the combination of MVA expressing VP2 and NS1 increased the immune protection conferred against a heterologous serotype of AHSV [68].

Epizootic hemorrhagic disease virus (EHDV) infects ruminants and causes severe disease mainly in deer [164]. An animal laboratory model would facilitate the studies and evaluation of vaccines against this virus. The IFNAR(−/−) mice has also been proposed as a mouse model to study EHDV infection with promising results [69]. Previously, the virus was shown to fatally infect newborn Swiss outbred mice after intracerebral inoculation [165]; however, newborn mice cannot be used for vaccination experiments.

The IFNAR(−/−) mice are susceptible to the infection with EHDV, in a dose-dependent manner. Animals displayed clinical signs similar to those observed in BTV-infected IFNAR(−/−) mice with the exception of conjunctivitis. A dose of 5 × 10^5^ PFUs killed all mice and they presented enlarged spleens and multiple necrotic foci in the liver as well as large amounts of EHDV RNA in spleen [69]. These are some of the organs where virus can be found in viremic deer (OIE 2014). More work is needed to continue characterizing aspects of the pathology of different serotypes of EHDV and to evaluate potential vaccines in the mouse model.

## 9. Conclusions

During the last decades, arboviruses have expanded their geographic range and caused an increasing number of outbreaks along all continents, enhanced by factors like climate warming, urbanization, global trade, travel, and changes in land uses [166]. Arboviruses incorporate a vast collection of genetically diverse viral pathogens. These viruses are peculiar as many of them are zoonotic and are transmitted by arthropod vectors, an added difficulty, being a serious harm to the society and animal welfare. In order to understand the arbovirus biology during infection and to develop an effective treatment against them, an adequate animal model for these studies is required. Mouse models deficient in IFN signaling are used to overcome the natural resistance of immunocompetent mice against non-mouse-specific viral infections, due to their inability to generate a complete immune response. Their use requires careful interpretation of results due to differences in the immunological state between wild-type and IFNAR(−/−) mice, and in the biology between mice and humans or large animals. However, there is no doubt about the utility of IFNAR(−/−) mouse models in the field of virology research, pathogenesis, immunobiology of the infections, arbovirus transmission, and vaccine testing.

The IFNAR knockout mice have served to study the role of some non-structural proteins as NSs of RFVF in the evasion of the type I IFN response, antagonizing IFN function. In this case, two attenuated RVFV strains with mutations in the NSs gene, *MP12* and clone 13, are highly virulent in IFNAR(−/−) mice, but remain attenuated in IFN-γ receptor-deficient mice and immunocompetent mice. The IFNAR(−/−) mouse model has also been used to study viral pathogenesis. In some cases, infection in this model leads with non-specific signs as ataxia or weight loss, or the severity of the infection is strain- and age-dependent as occur with ZIKV infections. In contrast, there are some examples that closely mimics hallmarks of natural host disease such as the case of CCFHV infections, where proinflammatory host responses, severe thrombocytopenia, and coagulopathy are observed; BTV infection, that leads to damage in lung and lymphoid organs and alteration in the level of blood parameters; or CHIKV, that exhibits a marked tropism for skeletal muscles, joints and skin, that constitute the classical symptomatology and organ affectation in the natural hosts. In some other cases, this model was useful to study various important phenomena of disease, as the role of type I IFN responses to control the access to the CNS, as the case of WNV, the study of the sexual and vertical transmission of ZIKV or the antibody dependent enhancement mediated by sub-neutralizing antibodies during secondary DENV infections.

For vaccine testing, the following animal model features are desirable: robust, reproducible viremia, immuno-competent, and pathology and clinical signs similar to those found in the host. Unfortunately, there is no model that fulfills all these criteria. The IFNAR(−/−) mice have defective innate immune responses, which can lead to limited adaptive immunity [20,167,168,169]. In contrast, many studies have shown the viability of this model to test vaccines and to study the adaptive response induced by them. They were able to trigger strong humoral and cellular immune responses comparable with those achieved in the immunocompetent model, as it has been shown in this review for RVFV, CCHFV, DENV, ZIKV, CHICK, BTV or AHSV, where high levels of neutralizing antibodies that block the virions or cytotoxic CD8 T cell responses, able to clear the infection, were induced using different platforms (inactivated vaccines, attenuated-replication defective, subunit vaccines, DNA or viral vector based vaccines) and vaccination strategies (single dose, prime-boost).

This review has summarized the characterization studies of relevant arboviruses in knockout out IFNAR mice to provide a small animal model for studying pathogenesis and control strategies. Experimental infections of IFNAR(−/−) mice with many of the studied arbovirus closely mimics hallmarks of these viruses in their natural hosts, although extrapolation of the results obtained must be done with care due to differences in the biology between mouse and humans or large animals and the immunosuppressed state of this model. Taking all these points together, the use of IFNAR(−/−) mice as a model to study arbovirus transmission, pathogenesis, virulence, and protective efficacy of new antiviral strategies and new generation marker vaccines has been widely demonstrated, being an adequate model in the initial steps of arbovirus research.

## Figures and Tables

**Table 1:** Summary of viruses that have used the IFNAR(−/−) mouse model to study pathology and vaccine efficacy.

9	Serotype or Strain	Mortality	Clinical Signs	Vaccine Model
Rift Valley fever virus [29,30,31,32,33]	ZH548, MP12, Clone 13	Yes	Swollen and congested liver, acute hepatitis. Ruffled fur, hunched posture, and lethargy	DNA-Gn/Gc
Crimean Congo Fever Virus [5,34,35,36,37,38,39,40]	IbAr 2000, IbAr 10200	Yes	Labored breathing and porphyry around the nostrils and eyes. Organ pathology (liver and lymphoid tissue), thrombocytopenia, coagulopathy, weight loss, ruffled fur, hunched posture, and lethargy.	CCHFV alum-adjuvanted vaccines, VLPs, DNA or viral vector vaccines (MVA and adenovirus) expressing nucleocapsid protein or glycoproteins
Schmallenberg virus [41,42,43]	wild-type SBV (wtSBV), isolate BH80/11	Partial (50%)	Weight loss, ataxia, and apathy.	DNA-Gn/Gc/N, DNA-N-terminal GC, recombinant-N-terminal GC
Dengue virus [44,45,46,47,48]	DENV-1 DENV-2 DENV-3 DENV-4	Yes Yes ND No	Severe dengue-like disease.	Live attenuated mutants in the 2′-O-methyltransferase (2′-O-MTase) of DENV-1 and DENV-2
Yellow fever virus [49]	Asibi or Angola73	Yes	Viscerotropic disease.	ND
Zika virus [50,51,52,53,54,55]	MP1751 H/PF/2013 MR 766. 5 ZIKV-Paraiba	Yes	Severe disease, including hind limb weakness and paralysis.	Vaccinia-based single vector encoding polyprotein DNA-prME
West Nile virus [28,56]	WNV strain 3000.0259	Yes	Hunched posture, ruffled fur and reduced activities. Encephalitis.	RepliVAX WN, single-cycle West Nile vaccine
Japanese encephalitis virus [57]	JaOArS982	Yes	Slow movement, ataxia, piloerection, anorexia and continuous weight loss.	ND
Chikungunya virus [58]	CHIKV-21	Yes	Weakness of the limbs (loss of muscle tone) and lethargy.	VSV-CHIKV-E3-E2-6K-E1 EILV/CHIKV chimeras
Sindbis virus [59]	TR339	Yes	Weight loss and fur ruffling.	ND
Venezuelan equine encephalitis [60]	V-3000	Yes	Pronounced hunching, lethargy, prostration, and death.	ND
Vesicular stomatitis virus [61,62]	VSV Indiana	Yes	Neuropathy.	ND
Thogoto virus [63,64]		Yes	Pathological lesions in the lungs, liver and intestine.	ND
Bluetongue virus [6,65,66]	BTV-1 BTV-2 BTV-4 BTV-8 BTV-16	Yes	Splenomegaly, congested lung. Hunched posture, ruffled fur, conjunctivitis.	DNA, Herpesvirus Poxvirus, Baculovirus, and bacterial expressed proteins, Adenovirus
African horse sickness virus [67,68]	AHSV-1 AHSV-3 AHSV-4 AHSV-9	Yes	Ruffled fur, lethargy, ocular discharges, hemorrhages in lung, splenomegaly, congestion of liver.	DNA, Poxvirus
Epizootic hemorrhagic disease virus [69]	EHDV-7	Yes	Splenomegaly, necrotic foci in the liver.	ND

* ND: Non-determined.

**Table 2:** Summary of Bluetongue virus (BTV) vaccine studies evaluated in IFNAR(−/−) mouse model.

Vaccine Based on	Protein Expressed	Protection against Homologous BTV	Protection against Heterologous BTV	Reference
BTV inactivated vaccine	-	Yes	Not determined	Calvo-Pinilla et al., 2009 [6]
MVA virus	VP2 and VP5	Partial	No	Calvo-Pinilla, 2009 [134]
Bovine herpes virus	VP2	Partial	No	Franceschi et al., 2011 [143]
Equine herpes virus	VP2 and VP5	Partial	No	Ma et al., 2012 [144]
MVA virus	VP2, VP5, and VP7	Yes	No	Calvo-Pinilla et al., 2009 [134] Jabbar et al., 2013 [145]
MVA virus	VP2, VP7, and NS1	Yes	Yes	Calvo-Pinilla et al., 2012 [135]
muNs microspheres	VP2, VP7, and NS1	Yes	Partial	Marín-López et al., 2014 [146]
Bacterial expressed proteins	VP2 domains	Yes	No	Mohd Jaafar et al., 2014 [147]
Adenovirus	VP2, VP7, and NS3	Yes	ND	Martín et al., 2015 [148]
muNS/MVA virus	VP2, VP7, and NS1	Yes	Yes	Marín-López et al., 2017 [149]
MVA virus	NS1	Yes	Yes	Marín-López et al., 2018 [150]
DNA/Fowlpox virus	VP2 and VP5	ND	ND	Li et al., 2015 [151]
Baculovirus expressed proteins	VP2 alone or fused to APCH	ND	ND	Legisa et al., 2015 [152]
Plant-produced protein	VP2 alone or VP2 B-cell epitope sequences	ND	ND	van Zyl et al., 2017 [153]
Bacterial and baculovirus expressed proteins	VP2, VP3, VP7, NS2, truncated VP5	ND	ND	Mohamed et al., 2018 [154]

* ND: Non-determined.
